# Correction: HPV16-Expressing Tumors Release Multiple IL1 Ligands to Orchestrate Systemic Immunosuppression Whose Disruption Enables Efficacy of a Therapeutic Vaccine

**DOI:** 10.1158/2159-8290.CD-26-0046

**Published:** 2026-03-02

**Authors:** Morgane Lecointre, Jérémy Guillot, Rachel Marcone, Dilara Ozdoganlar, Marjorie Cayatte, Elin Jaensson Gyllenbäck, David Liberg, Nadine Fournier, Krisztian Homicsko, Douglas Hanahan

In the original version of this article (1) the color code in Fig. 4C (left) and Supplementary Fig. S4D (left) is reversed. These panels are now corrected to red labeling for upper 25% and teal labeling for lower 25%. The authors regret this error. The error has been corrected in the latest online HTML and PDF versions of the article.
